# Limitations of Improved Nitrogen Management to Reduce Nitrate Leaching and Increase Use Efficiency

**DOI:** 10.1100/tsw.2001.457

**Published:** 2001-12-11

**Authors:** James L. Baker

**Affiliations:** Department of Agriculture and Biosystems Engineering, Iowa State University, Ames, USA

**Keywords:** BMPs, nutrient management, runoff, mixing zone, wetlands, tillage, cropping

## Abstract

The primary mode of nitrogen (N) loss from tile-drained row-cropped land is generally nitrate-nitrogen (NO_3_-N) leaching. Although cropping, tillage, and N management practices can be altered to reduce the amount of leaching, there are limits as to how much can be done. Data are given to illustrate the potential reductions for individual practices such as rate, method, and timing of N applications. However, most effects are multiplicative and not additive; thus it is probably not realistic to hope to get overall reductions greater than 25 to 30% with in-field practices alone. If this level of reduction is insufficient to meet water quality goals, additional off-site landscape modifications may be necessary.

## INTRODUCTION

The issue of nitrate-nitrogen ($NO_3$-N) leaching and the resultant contamination of surface and groundwater resources is a continuing public concern. $NO_3$-N can pose human health hazards as well as cause ecological damages such as hypoxia in the Gulf of Mexico[1]. A variety of means are available to reduce nitrate leaching, and they are the subject of this paper, primarily with respect to corn and soybean row-crop agriculture common in the Midwestern U.S. Corn Belt, where a major portion of the land has artificial subsurface (or "tile") drainage, a factor that has significant water quality impact[2].

Before considering management practices to reduce $NO_3$-N leaching losses, two points should be made. The first is that for economic corn (*Zea mays* L.) production, where a yield of at least 150 bu/acre would be expected, the amount of N needed for grain (~120 kg/ha), above-ground stover (~90 kg/ha), and roots (~60 kg/ha) totals about 270 kg/ha. Given that the corn plant might transpire 45 cm of water in a growing season, the ratio of $NO_3$-N to water taken up is 60 mg/l — quite a large number relative to levels of concern (e.g., the drinking water standard in the U.S. is 10 mg/l). That U.S. corn producers generally only supply a little over half the crop N needs as inorganic fertilizer and/or manure, with the other half having to come from "recycled" N in the soil and crop residue, also has implications for management practices to reduce $NO_3$-N leaching.

The second point is that a 50 bu/acre soybean (*Glycine max* L.) yield removes about 200 kg N/ha in the harvested grain; this is more than the soybean plant might fix from the atmosphere in a growing season[3]. Therefore, the "N credit" often given for corn following soybeans is due not to additional N fixed by the soybean plant, but to increased availability of N remaining in the soil and crop residue. Furthermore, when considering a total N mass balance for a corn-soybean system, depending on inputs from precipitation and animal manures that are recycled to the land vs. losses due to denitrification and leaching, it is not inconceivable in the Corn Belt that we are near a balance (with soil organic matter content stabilized to a new, lower, but constant value, compared to previous prairie or wetland conditions).

In considering management practices to reduce $NO_3$-N leaching, we realize leaching loss is a product of $NO_3$-N concentration and the volume of water drained. Fig. 1 illustrates a portion of the hydrologic cycle, with emphasis on the surface soil and the effect that the rate and route of infiltration have on $NO_3$-N concentrations and losses. Because $NO_3$-N is very soluble and not adsorbed to soil, it moves readily with water. The surface layer of soil that interacts with and releases chemicals to rainfall and surface runoff is fairly thin (shown as 1 cm in Fig. 1), and if that surface soil has good structure and is not already saturated or compacted, sufficient infiltrating water will move through it before runoff begins to move a significant portion of the $NO_3$-N present to a depth where it can not be lost with runoff. That is why $NO_3$-N concentrations in surface runoff from row-crop lands in the Corn Belt are usually in the 2 to 5 mg/l range; whereas, in subsurface drainage water from the same lands, $NO_3$-N concentrations are usually in the 10 to 20 mg/l range. The location of $NO_3$-N within soil aggregates, vs. near zones of preferential and higher water movement, will have an impact on $NO_3$-N concentrations in leaching water (this will be discussed further in the next section relative to tillage and also N fertilizer placement). Obviously, the rate of infiltration vs. the precipitation intensity will determine the volume of runoff, and the volume of infiltration, minus the capacity of the soil to store water, will determine the leaching volume.

## MANAGEMENT PRACTICES TO REDUCE NITRATE LEACHING

In-field management practices consist of those related strictly to the source or concentration term in the loss equation (such as the rate, method/placement, form/additives, and timing of N applications) and those related to both the concentration and transport, or volume of drainage, terms (such as tillage and cropping). In the following section, each of these management practice decisions will be evaluated as to their potential to reduce $NO_3$-N leaching (usually with the assumption that efficiency of use will increase concurrently).

### Rate

The rate of N application has a very direct effect on $NO_3$-N concentrations in subsurface drainage water. In some early work in Minnesota, Gast et al.[4] measured $NO_3$-N concentrations and losses with tile drainage from plots in continuous corn that received N at 20, 112, 224, and 448 kg/ha/year for 3 years. There was no effect of differential fertilization on $NO_3$-N concentrations during the first year, but for the second year, by rate, concentrations averaged 19, 25, 37, and 65 mg/l, respectively; corresponding numbers for the third year were 19, 23, 43, and 81 mg/l. Soil sampling at the end of the third year showed a buildup of $NO_3$-N in the 0- to 3.0-m soil profile for the two highest rates, with 425 and 770 kg/ha present for the 224 and 448 kg/ha fertilization rates, respectively. In more recent work in Minnesota, Randall and Mulla[5] reported that $NO_3$-N losses in tile drainage water from continuous corn plots increased from 8 kg/ha/year, with no N fertilizer applied, to 21 and 29 kg/ha/year when 134 and 202 kg/ha fertilizer N, respectively, were applied in the spring; corresponding numbers from fall N fertilization were 30 and 38 kg/ha/year.

In an early Iowa study, Baker and Johnson[6] found, in a corn-soybean-corn-oats rotation with N applied at 95 kg/ha in the corn years, that $NO_3$-N concentrations in tile drainage averaged 20.1 mg/l. When the N rate on an adjacent tile-drained plot was increased to 245 kg/ha, the concentrations averaged 40.5 mg/l. Concentration vs. time/flow-volume data showed there was a lag of about 1 month and 10 cm of flow before differential fertilization affected $NO_3$-N concentrations in tile drainage, and maximum concentrations were observed in the years following the years of large N fertilizer applications. Later work by Baker and Melvin[7] has shown that for a site in north-central Iowa, corn yields increased with N fertilizer rate for both continuous

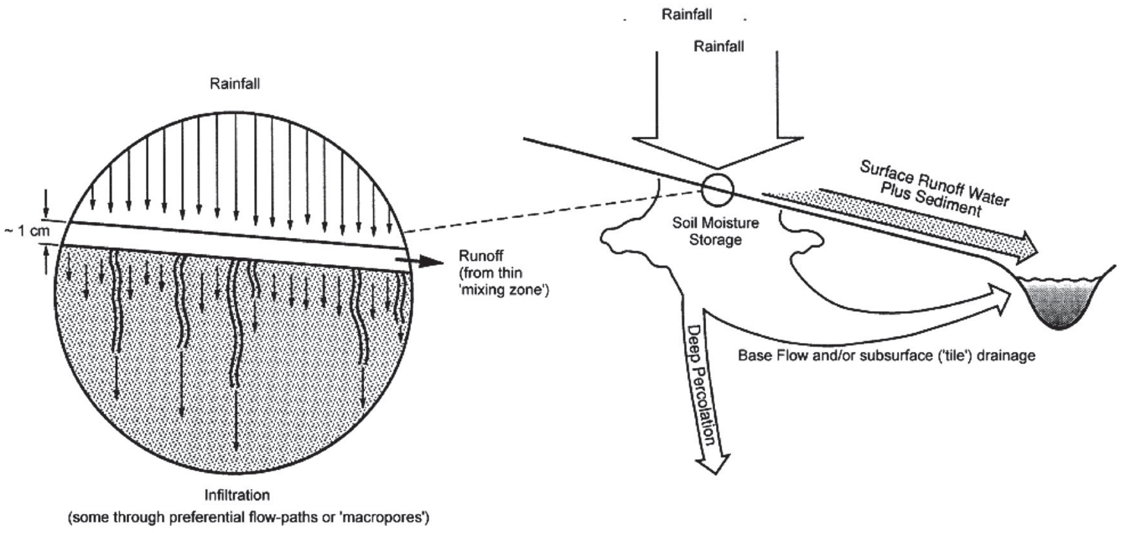

**FIGURE 1.** Schematic illustrating transport nodes and importance of "mixing zone."

corn (economic optimum N rate of about 200 kg/ha) and corn rotated with soybeans (optimum of about 150 kg/ha). However, as shown in Table 1, $NO_3$-N concentrations in tile drainage from the treatment plots also increased with N application rate and were well above 10 mg/l at the optimum fertilization rates. Phase-two follow-up work on rate and method of N application has occurred on the same plots, and the resultant concentration data in Table 2 again show the effect of increasing $NO_3$-N concentrations with increasing N application rates. For the corn-soybean rotation for both phases of this work, the effect of differential N fertilization in the corn year was more evident in the following year when soybeans were grown with no N applied.

**TABLE 1**
**Corn Yields and $NO_3$-N Concentrations in Tile Drainage as Affected by N Application Rate (1990–1993)**

| Rotation | N Rate (kg/ha/year) | Corn Yield (bu/ac) | $NO_3$-N Concentration (mg/l) |
|---|---|---|---|
| Continuous corn | 0 | 55.5 | 5.0 |
| | 56 | 76.2 | 8.3 |
| | 112 | 93.2 | 9.1 |
| | 168 | 114.3 | 13.2 |
| | 224 | 116.2 | 15.5 |
| Corn-soybeans | 0 | 80.2 | 8.5 |
| | 56 | 104.2 | 9.9 |
| | 112 | 132.5 | 10.7 |
| | 168 | 136.1 | 11.7 |

**TABLE 2**
**Corn Yields and $NO_3$-N Concentrations in Tile Drainage as Affected by Rate and Method of N Application (1995–1999)**

| Rotation | Method[a] | N Rate (kg/ha/year) | Corn Yield (bu/ac) | $NO_3$-N Concentration (mg/l) |
|---|---|---|---|---|
| Continuous corn | LCD | 135 | 94.1 | 10.3 |
| | | 180 | 110.6 | 12.3 |
| | PIFA | 135 | 89.1[b] | 8.4 |
| | | 180 | 86.9[b] | 13.2 |
| | Knife | 135 | 98.6 | 8.9 |
| | | 180 | 107.7 | 17.0 |
| Corn-soybeans | LCD | 45 | 104.4 | 8.9 |
| | | 90 | 111.9 | 8.3 |
| | | 135 | 122.1 | 10.7 |
| | PIFA | 45 | 92.7 | 6.5 |
| | | 90 | 103.5 | 7.1 |
| | | 135 | 122.8 | 7.3 |
| | Knife | 45 | 105.2 | 5.9 |
| | | 90 | 117.4 | 8.1 |
| | | 135 | 125.0 | 11.9 |

[a] LCD stands for localized compaction and doming applicator, PIFA stands for the point injection fertilizer applicator used with ridge tillage, and knife stands for the conventional knife applicator.
[b] Corn yields were lower because of weed control problems.

## Method/Placement

The method of application or placement of applied N is receiving increased attention because the location in/within the soil relative to zones of higher water movement influences the degree of anion (including $NO_3$-N) leaching. In a rainfall simulation study of water and anion movement under ridge tillage[8], $NO_3$-N and bromide (Br) placed in the elevated portion of the ridge had reduced leaching, compared to a similar application with flat tillage. After 7.2 cm of rain a day after anion application, 89 and 94% of the applied $NO_3$-N and Br, respectively, were recovered by soil sampling the top 1.2 m of the soil profile; corresponding numbers for flat tillage were 53 and 62%. Visual observation and water content measurements showed that more water infiltrated in the valleys between the ridges than into the ridges (where the anions had been applied), because some water ran off the ridge slopes and ponded in the valleys. Kiuchi et al.[9] measured the effects of different subsurface barriers, including plastic disks and compacted soil, on anion leaching in soil columns. All barriers placed over applied chloride (Cl, with cross-sectional areas $\leq$8% of the column cross-sectional area) delayed column breakthrough and reduced peak concentrations of Cl. In a follow-up study, Baker et al.[10] measured Br leaching from undisturbed blocks of soil, where the Br was broadcast applied or point injected with and without compaction around the point of injection. Compaction significantly reduced Br leaching, with concentrations for that treatment on no-till blocks of soil being 7 and 11% of the uncompacted point injection and broadcast application treatments, respectively; corresponding numbers for chisel plow were both 15%.

As a result of these studies, a new fertilizer applicator[11] has been designed, constructed, and tested to place N in an environment that impedes excessive water movement; two patents have been received for this applicator (Pat. No. 5,792,459 and 5,913,368). Comparison of $NO_3$-N movement for N applied with the localized compaction and doming applicator (LCD ) with that applied with a conventional knife application during the corn growing season showed that the average depth of leaching for the LCD was only 60% of that for the knife. In another field study[12], soil was sampled to 0.8 m, 83 days after $NO_3$-N and Br were applied at about 135 kg/ha each with both LCD and knife applicators. This 83-day period in 1993 was wetter than normal, and there was about 25 kg/ha more of both $NO_3$-N and Br retained in the sampled soil for the LCD vs. the knife applicator. In the following year, 1994, precipitation was slightly below normal, and application method had no effect on either $NO_3$-N or Br recovered in soil sampled 68 and 131 days after application. In a lysimeter study[13], three fluorbenzoate tracers were used to compare leaching (to the 1.2-m-deep drainage collection tube) of these anions applied surface broadcast, with a conventional knife applicator, and with the LDC applicator. At the end of 6 months, leaching losses were 4, 5, and 1% of that applied by the three methods, respectively; corresponding numbers after 18 months were 17, 25, and 13%. In an on-going field study[7], $NO_3$-N concentrations in subsurface drainage and corn yields are being measured for different methods of N application (see Table 2 for 5-year averages). One application method is use of a point-injector fertilizer applicator[14] (PIFA), developed at Iowa State University, in conjunction with ridge tillage. Although differences in average $NO_3$-N concentrations were generally not large, there was a trend at the highest (and economic optimum) N rates for both rotations, for the knife to have the highest concentration (statistically significant for continuous corn).

## Timing

Better timing of N application(s) relative to crop needs reduces the opportunity for $NO_3$-N leaching[15]. The corn plant's need for N is not that great until at least 4 weeks after plant emergence, which generally means the greatest uptake period is mid-June through July. Fall application, while sometimes having advantages in the way of N pricing or time to do field work, exposes the applied N to leaching losses over an extended period. Randall and Mulla[5] reported that average annual $NO_3$-N leaching loss from continuous corn, for N applied in the fall at 134 kg/ha, was 30 kg/ha; whereas it was 21 kg/ha when applied in the spring. Corresponding numbers for a 202 kg/ha N application rate were 38 and 29 kg/ha. Besides reducing $NO_3$-N leaching in this 5-year study, spring application increased corn yields by about 10%. Randall and Mulla[5] also reported that, across a 4-year flow period, annual average $NO_3$-N concentrations in tile drainage water from corn plots receiving 150 kg N/ha in late fall, late fall plus nitrapyrin, spring preplant, and split (40% preplant and 60% sidedress) were 20, 17, 16, and 16 mg/l, respectively.

In a 3-year study of tillage and split N application effects on $NO_3$-N in tile drainage water[16], there were no treatment effects the first year, and there was essentially no tile flow the second year (see Table 3). In the third year, for no-till continuous corn, a split-application lower-rate treatment (125 kg N/ha — split 25, 50, 50 at planting, 20 days later, and another 20 days later) produced average $NO_3$-N concentrations of 11.4 mg/l, significantly lower than the 14.7 mg/l for 175 kg/ha (all applied at planting).

## Form/Additives

Because of soil adsorption of ammonium-nitrogen ($NH_4$-N), additions of ammonical N (or N that will form $NH_4$-N) will significantly reduce the N leaching potential for the time the N stays in the $NH_4$-N form. One approach to extend the "life" of $NH_4$-N is to add a nitrification inhibitor, such as nitrapyrin, to the ammonical-N being applied to reduce the conversion rate to $NO_3$-N. Randall and Mulla[5] reported, for a 4-year study, that $NO_3$-N concentrations in tile drainage where anhydrous ammonia was fall-applied for corn at 150 kg N/ha were 20 mg/l; when nitrapyrin was added to the anhydrous ammonia, the concentration was 17 mg/l.

## Tillage

The degree of tillage has the potential to affect both $NO_3$-N concentrations and the volumes of surface and subsurface drainage, where tillage can range from complete inversion with the moldboard plow to no tillage at all. Mineralization of N in soil organic matter and crop residue will affect the amount of $NO_3$-N available for leaching, and increased aeration of surface soils with increased tillage is expected to increase mineralization. Furthermore, the destruction of structure, including macropores, in

**TABLE 3**
**Corn Yields and $NO_3$-N Concentrations in Tile Drainage as Affected by Tillage and Rate/Timing of N Application (1984–1986)**

| Tillage | Timing | N Rate (kg/ha) | Corn Yield (bu/ac) | $NO_3$-N Concentration (mg/l) |
|---|---|---|---|---|
| *1984* | | | | |
| Conventional | Preplant | 175 | 130 | 10.7 |
| No-till | Preplant | 175 | 129 | 11.1 |
| No-till | Split[a] | 125 | 132 | 11.8 |
| *1985* | | | | |
| Conventional | Preplant | 175 | 113 | —[b] |
| No-till | Preplant | 175 | 118 | 11.7 |
| No-till | Split | 125 | 119 | —[b] |
| *1986* | | | | |
| Conventional | Preplant | 175 | 179 | 23.2 |
| No-till | Preplant | 175 | 170 | 14.7 |
| No-till | Split | 125 | 165 | 11.4 |

[a] Split was 25, 50, and 50 kg/ha at planting, 20 days later, and another 20 days later.
[b] There was no tile flow for these treatments.

surface soil with tillage affects both the rate and route of infiltrating water[17]. The tillage system used also influences the options available for N application; in particular, the degree of incorporation possible decreases with the decreased severity of tillage.

Several studies have been performed where the combined effects of tillage[18] have been measured in terms of $NO_3$-N concentrations and losses in tile drainage from crops produced with different tillage systems. In one extensive 3-year study in northeast Iowa[19], average $NO_3$-N concentrations in tile drainage water were measured as a function of crop rotation and tillage. As shown in Table 4, concentrations for no-till flat and ridge tillage were lowest of the four tillage systems studied, and moldboard plow was the highest. When concentration data were combined with flow volume data to calculate losses, somewhat lower flows with the moldboard plow system partially offset the higher concentrations; losses for no-till and ridge-till were less than for moldboard plow, which in turn, were lower than for chisel plow for the corn-soybean and soybean-corn rotations. For continuous corn, the order was moldboard plow less than ridge-till less than no-till less than chisel plow. The lower concentrations with no-till are believed to be due to less mineralization with no soil disturbance, movement of a greater percentage of water through preferential flow-paths (possibly "by-passing" some of the N in the no-till soil profile), and possibly some dilution due to higher average infiltration rates and drainage volumes with no-till. Data in Table 3 for the central Iowa study noted earlier show that average $NO_3$-N concentrations for the moldboard system were higher than for no-till when the same N treatment, 175 kg/ha applied preplant, is considered. In an 11-year study with continuous corn in Minnesota, $NO_3$-N concentrations from no-till plots receiving 200 kg/ha/year averaged 13 mg/l; for moldboard plow plots, the value was 15 mg/l[20].

**TABLE 4**
**$NO_3$-N Concentrations in Tile Drainage as Affected by Crop Rotation and Tillage (1990–1992)**

| Rotation | Moldboard Plow | Chisel Plow | Ridge-Till | No-Till |
|---|---|---|---|---|
| | (mg $NO_3$-N/l) | | | |
| Continuous corn | 36.9 | 31.0 | 21.7 | 21.9 |
| Rotation corn | 23.6 | 20.1 | 15.5 | 14.4 |
| Rotation soybeans | 16.4 | 16.2 | 13.4 | 12.1 |

[a] From Reference [19].

## Cropping

Much of the U.S. Midwestern Corn Belt is in a corn-soybean rotation, with much less continuous corn under the latest USDA Farm Program. Data in Tables 1, 2, and 4 show, depending on the amount of N applied but giving "credit" (usually 40 to 50 kg N/ha) for soybeans in the crop rotation, that $NO_3$-N concentrations in subsurface drainage for the corn-soybean rotations are less than or equal to those for continuous corn. The study by Baker and Melvin[7] also included continuous alfalfa, where the $NO_3$-N concentrations averaged about 5 mg/l, compared to the values above 10 mg/l shown in Table 1 for fertilized corn. In trying to establish alfalfa in the first year of that study, some plots remained fallow, and with soil N mineralization without plant uptake, the $NO_3$-N concentrations for those plots exceeded all others including continuous corn receiving 224 kg N/ha. In the 4-year Minnesota study on timing of N fertilization cited earlier[5], the average $NO_3$-N concentration in tile drainage from the fallow plots was 36 mg/l; the average for the four N fertilizer treatments (150 kg N/ha/year at different times) was 17 mg/l.

In a 4-year Minnesota study of the effect of crop system on average $NO_3$-N concentrations and losses in tile drainage water[21], $NO_3$-N concentrations were 32, 24, 3, and 2 mg/l, respectively, for continuous corn, corn-soybeans, alfalfa, and CRP (conservation reserve program with a grass-alfalfa mix). Because of higher flow volumes from row-crop plots, $NO_3$-N losses were 30 to 50 times higher than from the perennial crops.

## SUMMARY

"Fine-tuning" in-field management practices relative to rate, method, timing, and form/additives of N applications has the potential to decrease $NO_3$-N concentrations and therefore leaching losses with subsurface drainage. Use of the late-spring-soil-nitrate test (LSNT) can help in determining the correct N rate for corn, and there is some potential that a new soil test for amino sugar N will improve the soil test over the current $NO_3$-N analysis. However, given the large N needs for corn and the close relationship between yield and available N, it is unlikely in most cases that N application rates can be adjusted downward more than 10 to 15% without significant economic loss of production. Additional improvements in method and/or timing of N applications as discussed should also reduce $NO_3$-N leaching losses, but overall, it is probably not realistic to expect changes in rate, method, timing, and form/additives to ever reduce losses more than 25%. Increased use of conservation tillage, particularly no-till, should, on average, reduce $NO_3$-N concentrations, but again, the effect will be limited, probably less than 15% overall. Choice of cropping can have a much bigger influence; however, economics currently dictates that, in much of the U.S. Corn Belt, row-crop agriculture consisting of corn and soybeans will continue to dominate.

If in-field practices are not sufficient to obtain the desired degree of $NO_3$-N loss reduction, then off-site practices will have to be considered. Use of constructed/reconstructed wetlands do have considerable potential to remove $N0_3$-N in subsurface drainage routed through them. Denitrification is the dominant removal process, and residence time, temperature, and oxygen levels determine the degree of removal[22].

| | | |
|---|---|---|
| Received: | August | 13, 2001 |
| Revised: | November | 15, 2001 |
| Accepted: | November | 15, 2001 |
| Published: | December | 11, 2001 |

## BIOSKETCH

**James L. Baker** is a professor in the Department of Agricultural and Biosystems Engineering at Iowa State University. He has a Ph.D. in physical chemistry from Iowa State University (1971) and a B.S. in chemistry for the South Dakota School of Mines and Technology (1966), comes from a farm/ranch background, and has 30 years of research experience on agricultural nonpoint/water quality issues. His research has been with farm management practices and equipment (e.g., a point-injector fertilizer applicator) that result in protection of our soil resource against erosion, efficient use of agricultural chemicals and energy resources, and improved water quality of agricultural drainage. His recent work has also included the effects on water quality of off-site practices involving permanent vegetation and wetlands. Awards and Honors received include the Joan Hodges Queneau Palladium Medal (National Audubon Soc./Am. Assoc. Eng. Soc.) in April 1989; Fellow, American Society of Agronomy, November 1995; and Hancor Soil and Water Engineering Award, ASAE, July 1996.
